# Assessment of knowledge, attitude and practice of first aid among taxi operators in a Kampala City Taxi Park, Uganda: A cross-sectional study

**DOI:** 10.1016/j.afjem.2021.10.007

**Published:** 2022-01-12

**Authors:** Nelson Ssewante, Godfrey Wekha, Moureen Namusoke, Bereta Sanyu, Ayub Nkwanga, Racheal Nalunkuma, Ronald Olum, Lawrence Katumba Ssentongo, Rachel Ahabwe, Sharon Esther Kalembe, Vanessa Nalwoga Nantagya, Joseph Kalanzi

**Affiliations:** a School of Medicine, College of Health Sciences, Makerere University, Kampala, Uganda; b School of Health Sciences, College of Health Sciences, Makerere University, Kampala, Uganda; c Department of Emergency Medicine, College of Health Sciences, Makerere University, Kampala, Uganda

**Keywords:** First aid, Taxi driver, Conductor, First responder, Uganda

## Abstract

**Introduction:**

Road traffic accidents are among the leading causes of death in Uganda. Taxi operators are at a high risk of RTA and can potentially be first responders. This study, aimed to assess knowledge, attitude, and practice of first aid among taxi operators in the new taxi park, Kampala Uganda.

**Methods:**

A descriptive cross-sectional study was conducted in 2021 among taxi drivers and conductors in the New Taxi Park, Kampala City, Uganda. A semi-structured questionnaire was used to collect quantitative data from taxi operators after informed consent. Chi-square or Fisher's exact test and logistic regression were performed in STATA 16 to assess the association between first aid knowledge and demographics. *P* < 0.05 was statistically significant.

**Results:**

A total of 345 participants, majority males (*n* = 338, 98%) aged between 18 and 45 years (76.5%), were recruited. Although 97.7% (*n* = 337) had heard about first aid, only 19.4% (*n* = 67) had prior first aid training. Overall mean knowledge score was 40.1% (SD = 14.5%), with 29.9% (*n* = 103) having good knowledge (≥50%). Participants who had witnessed more than five accidents (aOR = 2.9, 95% CI = 1.7–4.8, *p* < 0.001), those with first aid kits (aOR = 1.7, 95% CI = 1.0–3.0, *p* = 0.38) were more likely to have good knowledge while those below post-secondary education level i.e., Primary (AOR = 0.2, 95% CI = 0.1–0.5, *p* ≤0.001) and secondary (aOR = 0.2, 95% CI = 0.1–0.6, *p* = 0.001), were less likely to have good knowledge. About 97% and 93% perceived first aid as important and were willing to undergo training, respectively; however, only 69% were willing to give first aid. Only 181(52.5%) had ever attended to accident victims.

**Conclusion:**

Majority of taxi operators had poor first aid knowledge. Factors associated with good knowledge included level of education, number of accidents witnessed, having first aid kits. Although their attitudes were favorable, practice was poor. Comprehensive training and refresher courses can help increase first aid knowledge, and improving practice.

## African relevance

• Low-and middle-income countries still face the challenge of underdeveloped emergency medical care services.
• Lay people are more than willing to participate in solving this problem.
• Comprehensive training of lay responders could serve as a cost-effective solution.

## Introduction

Globally, approximately 1.3 million people die as a result of road traffic crashes, with up to 50 million people suffering lifelong disabilities from nonfatal injuries. The heaviest burden of up to 93% of these fatalities occurs in low- and middle-income countries [1]. Uganda has one of the highest annual incidences of Road Traffic Accidents (RTA) in Sub-Saharan Africa. According to World Health Organisation (WHO), there were 12,036 RTA-related deaths in Uganda in 2016 alone, accounting for 4.5% of the total deaths in the country. Uganda also ranks 15 worldwide in terms of RTA-related deaths [2].

First aid is the immediate assistance provided to a sick or injured person until professional help arrives to preserve life, alleviate suffering, prevention of further illness or injury, and promoting recovery [3]. Studies show that even an untrained bystander attending to accident victims improves survival from grievous injuries and cardiac arrest 4., 5.. Any delays in detecting and providing care for RTA victims increase the severity of injuries 6., 7.. Improving postcrash outcomes requires not only access to quality hospital care but also timely prehospital care 1., 6.. In a study conducted in Canada, it was estimated that the survival rate can be raised from 8% to 32% if all bystanders provide first aid [8]. However, there is a paucity of skilled bystanders in Sub-Saharan Africa ready to offer first aid care to victims [6]. Additionally, nontrained bystanders often find it difficult to initiate first aid care in emergencies due to lack of self-confidence, fear of causing more harm to the victim, and medical-legal issues that may arise in the attempt to attend to the victims 9., 10.. To address these challenges, upscaling of bystander training must be prioritized.

In Uganda, the road sector is the most important mode of transportation, carrying up to 99% of the passenger traffic and 97% of freight cargo [11]. Taxi operators, including drivers and conductors, work mainly on passenger delivery service vans transporting people from one location to another. Taxis are among the most commonly involved means of transport in Uganda [12]. Therefore, taxi operators have more exposure to RTAs and therefore represent an important group of bystanders that, if skilled, may play an important role in curbing the high burden of fatalities, loss of property, and associated traffic injuries. However, this group has also been ignored by many countries including Uganda.

Moreover, no study had been conducted before to assess their knowledge and determine their role in the provision of prehospital services to accident victims. This study, therefore, aimed at assessing the knowledge, attitude, and practice of first aid among taxi operators in the new taxi park located in Kampala, Uganda.

## Methods

### Study design and area

This was a cross-sectional quantitative study in the New Taxi Park, located in Kampala, Uganda, in the year 2021. Kampala is the capital and the largest city in Uganda. It has two main taxi parks; the Old and New taxi parks with other small parks distributed all over the city's five divisions (Central, Kawempe, Makindye, Nakawa, and Lubaga). A total of about 16,000 taxis operate within the capital [12]. At the time this study was developed and conducted, only the New taxi park was operational with the Old taxi park under renovation. Taxis in this context are minibuses (locally known as Kamunye) with a maximum passenger carrying capacity of 14. They are privately owned and may operate within the city's premises or transport passengers for longer distances upcountry. They work parallel to the bus services in the passenger transportation business.

### Study population

Taxi drivers and conductors who were licensed to operate within the new taxi park.

### Selection criteria

All registered taxi drivers and conductors (taxi fare collectors) 18 years of age and above operating from any of the stages within the New Taxi Park were eligible for the study. Each driver was required to present a driver's license before enrollment in the study and these identified their respective conductors to minimize selection bias. Those who were out of reach at the time of this survey were excluded.

### Sample size and sampling procedure

The sample size was calculated using Kish Leslie's formula for cross-sectional studies

$$N=\frac{Z^{2}\mathrm{PQ}}{I^{2}}$$

where; N = Estimated sample size, Z = Standard normal deviated at 95% confidence interval corresponding to 1.96, P = Expected prevalence (0.5) since this was the first study in this population, Q = 1-P, and I=Precision of estimate (0.05). A total of 384 participants was obtained from this calculation.

### Data collection

Quantitative data was collected between March and April 2021 using pretested interviewer-administered semi-structured questionnaires with both open-ended and closed-ended questions to assess the participant's knowledge, attitude, and practice of first aid services in the study area. This questionnaire consisted of 15 questions on participants' characteristics, 8 questions on knowledge, 3 questions for attitude, and 5 on practice. Data was collected by trained medical students as research assistants.

### Data analysis

Upon completion of data collection, cleaning and entry were done using Microsoft Excel 2016 and exported to STATA 16.0 (College Station, Texas, USA) for analysis. Demographic characteristics, knowledge, attitude, and practice of the participants towards first aid were first summarized as frequencies and percentages for categorical variables, and mean/median for numerical variables. Knowledge score was calculated by awarding a point for every correct response to questions on knowledge with a maximum score of 20 points and converted to percentages. Knowledge was then dichotomized into good (≥50%) or poor knowledge (<50%). Chi-square or Fisher's Exact test and logistic regression were performed to assess the association between knowledge on first aid and demographics. *P* < 0.05 was considered statistically significant.

### Ethical consideration

This study was approved by the Mulago Hospital Research and Ethics Committee under reference number MHREC 2025. Administration clearance to access the Taxi Park was acquired from the KCCA department of Public Health and Environment. Verbal permission was obtained from the Chairperson of the taxi park before data collection. Participation in this study was voluntary upon explaining the study objectives, anticipated risks, and potential benefits on a one-on-one basis with participants, written consent forms were signed.

## Results

### Characteristics of participants

A total of 345 participants were interviewed and all their responses were included in the analysis. Of these, 98.0% (*n* = 338) were males with the majority being in the age range of 18–45 years (*n* = 264, 76.5%). More than half of the participants (*n* = 232, 67.3%) were married and 57.1% (*n* = 197) reported to have attained at least a secondary level of formal education. Half of the participants (*n* = 182, 52.8%) had a working experience of more than 10 years with up to 40.6% (*n* = 140) reporting to have witnessed more than 5 fatal road traffic accidents annually.

Of the 345 participants, 80.6% (*n* = 278) had never received any form of training in first aid. Up to 38.8% (*n* = 134) did not have first aid kits within their passenger vans. Table 1 summarizes the characteristics of participants.

**Table 1:** Characteristics of the participants and the factors associated with good first aid knowledge.

Variable (*N* = 345)	Frequency n (%)	Knowledge on first aid		P
		Good: n (%)	Poor: n (%)	
Overall		103 (29.9)	242 (70.1)	
Age				
18–30	114 (33)	36 (46.2)	78 (68.4)	0.799
31–45	150 (43.5)	42 (28)	108 (72)	
More than 45 years	81 (23.5)	25 (30.9)	56 (69.1)	

Sex				
Female	7 (2)	2 (28.6)	5 (71.4)	0.940
Male	338 (98)	101 (29.9)	237 (70.1)	

Marital status				
Cohabiting	30 (8.7)	1 (3.3)	29 (96.7)	0.001
Divorced	8 (2.3)	2 (25)	6 (75)	
Married	232 (67.3)	82 (35.3)	150 (64.7)	
Single	73 (21.2)	18 (24.7)	55 (75.3)	
Widowed	2 (0.6)	0 (0)	2 (100)	

Level of education				
Below primary level	5 (1.5)	3 (60)	2 (40)	0.001
Post-secondary	26 (7.5)	16 (61.5)	10 (38.5)	
Primary	117 (33.9)	28 (23.9)	89 (76.1)	
Secondary	197 (57.1)	56 (28.4)	141 (71.6)	

Occupation				
Conductor	82 (23.8)	25 (30.5)	57 (69.5)	0.886
Driver	263 (76.2)	78 (29.7)	185 (70.3)	

Work experience				
10 years and above	182 (52.8)	50 (27.5)	132 (72.5)	0.580
5 to 9 years	88 (25.5)	28 (31.8)	60 (68.2)	
Less than 5 years	75 (21.7)	25 (33.3)	50 (66.7)	
Daily working time				
10 h and above	168 (48.7)	55 (32.7)	113 (67.3)	0.175
6 to 9 h	155 (44.9)	45 (29)	110 (71)	
Less than 5 h	22 (6.4)	3 (13.6)	19 (86.4)	

Sleeping period				
6–8 h	174 (50.4)	53 (30.5)	121 (69.5)	0.880
<6 h	151 (43.8)	45 (29.8)	106 (70.2)	
>8 h	20 (5.8)	5 (25)	15 (75)	

Estimated number of accidents witnessed				
1 to 5	205 (59.4)	43 (21)	162 (79)	0.000
>5	140 (40.6)	60 (42.9)	80 (57.1)	

Ever heard of first aid				
No	8 (2.3)	3 (37.5)	5 (62.5)	0.700
Yes	337 (97.7)	100 (29.7)	237 (70.3)	

Prior first aid training				
No	278 (80.6)	83 (29.9)	195 (70.1)	0.999
Yes	67 (19.4)	20 (29.9)	47 (70.1)	

Has first aid kit				
No	134 (38.8)	32 (23.9)	102 (76.1)	0.053
Yes	211 (61.2)	71 (33.6)	140 (66.4)	

Awareness of the emergence response number				
No	177 (51.3)	53 (29.9)	124 (70.1)	0.971
Yes	168 (48.7)	50 (29.8)	118 (70.2)	

### Knowledge of first aid

Of the 345 participants, 86.7% (*n* = 299) were aware that first aid should be initiated at the scene and 84.1% (*n* = 290) were aware that it should be initiated by bystanders. However, a significant percentage were not able to identify any sign of airway compromise (*n* = 104, 30.1%), maneuvers that can be used to open blocked airways (*n* = 306, 88.7%), or the suitable position for victims with airway compromise (*n* = 121, 35.1%). Application of tourniquets (*n* = 172, 49.9%) and application of pressure and dressing (n = 172, 49.9%) were identified as first aid methods of arresting bleeding, while application of a splint (*n* = 225, 65.2%) was identified by the majority as the first aid for the broken limbs (Table 2). Profuse bleeding (*n* = 262, 75.9%), Fractures (*n* = 223, 64.6%), loss of consciousness (*n* = 218, 63.2%), and traumatic wounds (*n* = 184, 53.0%) were identified as the main indications for hospital care. Other indications frequently mentioned included difficulty in breathing, severe pain, and vulnerable groups; children, women, and elderly.

**Table 2:** Knowledge on first aid among taxi drivers and conductors in Kampala.

Variable	Frequency	%
Mean knowledge score = 40.1% (SD = 14.5%)		
Area of first aid initiation		
As soon as the victim reaches the hospital	33	9.6
Don't know	13	3.8
Immediately at the scene	299	86.7
Who initiates first aid?		
Bystander	290	84.1
Don't know	16	4.6
Health worker	39	11.3
Knowledge of first aid airway maneuvers		
Signs of airway compromise		
Fast breathing	144	41.7
Noisy breathing	86	24.9
Slow breathing	120	34.8
No breathing	74	21.5
Don't know	104	30.1
Mention any airway manoeuvre		
Jaw thrust	4	1.2
Chin lift with head tilt	2	0.6
Recovery position	34	9.9
Don't know	306	88.7
How do you position the victim		
Don't know	121	35.1
Place the victim face up	192	55.7
Place the victim sideways	32	9.3
First aid for bleeding		
Applying a tourniquet	172	49.9
Apply pressure and dress	172	49.9
Apply alcohol	34	9.9
Lift the injured part above the body level	8	2.3
Don't know	62	18.0
First aid for broken limb		
Apply a splint	225	65.2
Don't know	65	18.8
Leave it open until victim is in hospital	55	15.9

The overall mean knowledge score was 40.1% (SD = 14.5%). Only 29.9% (*n* = 103) of the participants had good knowledge. Knowledge score was significantly high among those who had witnessed more than five accidents compared to those who had witnessed 1 to 5 accidents (43.9% vs 37.6%) and those who had a first aid kit (42.1% vs 36.9%). Other factors that influenced first aid knowledge included marital status and level of education and emergency response first contact persons as shown in Table 1.

At bivariate analysis, first aid knowledge was significantly associated with marital status (*p* = 0.001), level of education (p = 0.001), and the number of accidents witnessed (*p* < 0.001). Having a first aid kit had a marginal association (*p* = 0.053). On multivariable logistic regression (Table 3), participants who had witnessed more than five accidents were 2.9 times more likely to have good first aid knowledge (aOR = 2.9, 95% CI = 1.7–4.8, *p* < 0.001), those with a first aid kit were 1.7 times more likely to have good knowledge on first aid (aOR = 1.7, 95% CI = 1.0–3.0, *p* = 0.38). Participants who stopped in primary (aOR = 0.2, 95% CI = 0.1–0.5, p < 0.001) and secondary (aOR = 0.2, 95% CI = 0.1–0.6, *p* = 0.001) were less likely to have good knowledge compared to those who attained postsecondary education.

**Table 3:** Multivariable logistic regression showing factors associated with good knowledge on first aid.

Variables	Adjusted odds ratio	95% confidence interval	P
Education			
Post-secondary	Reference		
Below primary level	2.0	0.0–0.6	0.517
Primary	0.2	0.1–0.5	<0.001
Secondary	0.2	0.1–0.6	0.001
Working hours			
10 h and above	Reference		
6 to 9 h	1.0	0.6–1.6	0.909
Less than 5 h	0.3	0.1–1.2	0.098
Number of accidents witnessed			
1 to 5	Reference		
>5	2.9	1.7–4.8	<0.001
Has a first aid kit			
No	Reference		
Yes	1.7	1.0–3.0	0.038

### Attitude towards first aid

**Fig. 1:**
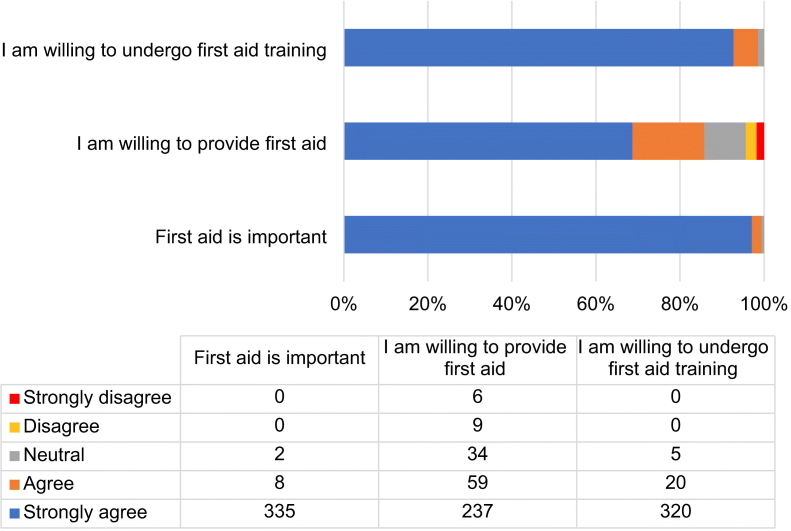
Attitudes of taxi drivers and conductors towards first aid.

Overall, participants had a good attitude towards first aid as shown in Fig. 1. Majority (*n* = 335, 97.1%) strongly agreed that first aid was an important aspect; none disagreed. Almost all (*n* = 340, 98.6%) were willing to undergo training to acquire first aid skills, only 1.4% (*n* = 5) were neutral, and none were against this training. However, although 85.8% (*n* = 296) were willing to provide first aid to the victims, up to 4.3% (*n* = 15) were against this practice and 9.9% (n = 34) were neutral.

### First aid practice

Overall, 37.1% (*n* = 128) of the participants had ever used a first aid kit before this survey, and a half (*n* = 181, 52.5%) had ever attended to an accident victim. Of those that had ever attended to an accident victim, 70.7% (n = 128) had attended to victims with active bleeding, 47.5% (*n* = 86) to victims with fractures with only 9.9% (n = 18) attending to victims with airway problems. Moreover, up to 8.1% (*n* = 28) did not know who to contact first for emergency rescue in case of need. Police were identified by the majority (*n* = 264, 76.5%) as the first point of reference they contact in emergency situations. More details on practices are provided in Table 4.

**Table 4:** Experiences and practices of taxi drivers and conductors with performing first aid.

Variable	Frequency	%
Utilisation of the first aid kit		
No	217	62.9
Yes	128	37.1
Emergence response contacts		
Ambulance	27	7.83
Don't know	28	8.12
Known doctor	26	7.54
Police	264	76.52
Ever attended to any victim		
No	164	47.5
Yes	181	52.5
First course of action (n = 181)		
Attended to the victim's injuries	123	68.0
Called ambulance/police	25	13.8
Took victim to the health unit	33	18.2
Victim had an airway problem (n = 181)		
No	163	90.1
Yes	18	9.9
Course of action (n = 18)		
Opened airway	4	22.2
Positioned victim for fresh air	11	61.1
Started chest compressions	3	16.7
Victim had active bleeding (n = 181)		
No	53	29.3
Yes	128	70.7
Course of action (n = 128)		
Applied pressure and dress	69	53.9
Applied tourniquet	36	28.1
Raised the limb to stop bleeding	23	18.0
Victim had a fracture (n = 181)		
No	95	52.5
Yes	86	47.5
Course of action (n = 86)		
Applied a splint	61	70.9
Immobilised the fractured limb	25	29.1

## Discussion

Uganda among other low- and middle-income countries (LMIC) bears the heaviest burden of Road Traffic Injuries (RTIs) with deaths due to RTIs at 28.9 per 100,000 population [13]. The deaths are mostly attributed to delays in access to simple life-saving interventions like control of bleeding, airway management, lack of skills, and ill equipment of first responders 6., 13.. This has greatly impacted the economy due to the loss of an economically productive population [14]. Our study showed a low level of knowledge but a positive attitude towards first aid among taxi drivers and conductors in Uganda.

In this study, we found out that only 29.9% of participants had good knowledge with an overall mean score of 40.1%. In a similar study conducted in Ethiopia, it was found that 50.35% of commercial taxi drivers had adequate knowledge but with an overall mean knowledge score of 39.7% [15]. These findings are almost similar to results from a study conducted in Nigeria where only 37.6% of commercial inter-city taxi drivers correctly identified basic resuscitation priorities [16]. In Zambia, the mean scores of taxi drivers and conductors respectively were 53.1% and 45.2% [17]. These findings communicate a big gap in knowledge of first aid among developing countries.

Factors significantly associated with first aid knowledge included; level of education, number of accidents witnessed, marital status, and availability of a first aid kit. These findings were in agreement with those of the Ethiopian study. However, contrary to what was expected and to the latter study, working experience and prior first aid training were not significantly associated with knowledge [15]. In Ethiopia, it was found that up to 26.8% of participants had prior first aid training while in our study only 19.42% had received training in their past. This much was far better compared to the study findings from India where only 1.2% of their participants had been trained [5]. An earlier study conducted in Uganda to assess the scalability of prehospital trauma care among lay first responders demonstrated that upscaling such training was associated with modest expenses [18]. This is further supported by studies exploring the role of lay responder training in Uganda which reported a significant increase in knowledge, and confidence in the practice of the acquired skills 19., 20., 21., 22., 23.. As demonstrated in Norway, the availability of trained bystanders positively impacted the patient's outcome [24]. Therefore, a priority shift towards bystander training is needed to address the emergency medicine challenges in resource-limited countries.

Attitude towards first aid was good and up to 98.5% felt that first aid was necessary. The majority of participants (94.6%) demonstrated a willingness to undergo training in First Aid. This has been the case in most studies where the majority of the participants had a good attitude. In most studies conducted in other parts of the world, participants were equally interested in undergoing first aid training 5., 15., 17.. This attitude amidst low levels of knowledge is likely due to the experience they get when they witness victims suffering or even dying at the accident scenes. This might spark the desire to equip themselves with the knowledge and skills to be able to save lives.

Our study revealed that half of the participants had attended to an accident victim in the past with up to 35.7% providing first aid at the scene. Others either called the police/ambulance or transported the victims to the nearest health unit. This was better compared to studies conducted in other countries with similar resource settings 5., 15., 16..

A possible explanation for the reported confidence in our participants given the limited training is the fact that Uganda has a low ambulance coverage, very few people are aware of the emergency response numbers with reports claiming that occasionally these numbers are either too busy or not available at all and delays in emergency response by the police. These factors coupled with the reported good attitude are likely to leave taxi operators with no choice but to intervene in emergencies. Simple maneuvers such as application of pressure and dressing of victims' wounds, splinting of fractured limbs were the interventions most reported by the participants in our study as well as in the aforementioned studies.

To the best of our knowledge, this is the first study to explore the knowledge, attitude, and practice of first aid among taxi drivers and conductors in Uganda. Though small in scale, findings from this study provide a potential use of available resources in a low-to-middle income country. Needless to say, this study suggests a great need for first aid training among taxi operators in the country. This can be achieved by reviewing licensing and public transport regulatory policies in the country to require all drivers and conductors have basic first aid training before acquisition of a taxi operating license. Such policies would ensure easy and quick access to affordable training services with reliable information. A collaboration between the Ministry of Works and Transport, and the Ministry of Health together with other stakeholders such as KCCA is important in this initiative.

Firstly, our major limitation was generalizability. This was mitigated to an extent by excellent comparison with similar studies conducted in other African countries as well as further abroad. Secondly, the level of first aid knowledge though well defined, may not necessarily correlate with true knowledge. This is because the cut-off point of 50% is somewhat subjective and is simply based on the limited number of questions asked. Therefore, it is conceivable that taxi operators are more or less knowledgeable than the study results suggest.

## Conclusion

The study found that majority of the taxi operators had inadequate knowledge of first aid. Knowledge was associated with the level of education, participant's marital status, number of accidents witnessed, and availability of first aid kits. Most of the respondents felt that first aid was necessary. However, their first aid practice was inadequate.

First Aid certificates should be a prerequisite for obtaining a taxi operating permit in Uganda and therefore, comprehensive training should be organized by stakeholders to ensure appropriate certification and renewal. Additionally, stakeholders including KCCA, Ministry of Works and Transport, and Uganda Police should ensure that all taxis are equipped with functional first aid kits for use in case of emergencies in addition to other safety requirements and that the public is made aware of who to call in an emergency.

## Funding

This research was supported by the Fogarty International Center of the National Institutes of Health, 10.13039/100000194U.S. Department of State's Office of the U.S. Global AIDS Coordinator and Health Diplomacy (S/GAC), and U.S. 10.13039/100009054President's Emergency Plan for AIDS Relief (PEPFAR) under Award Number 1R25TW011213. The content is solely the responsibility of the authors and does not necessarily represent the official views of the National Institutes of Health.

## Dissemination of results

Results from this study were shared with Health professional education and partnership initiative (HEPI), Kampala Capital City Authority (KCCA) the governing body of Kampala City, and the Ministry of Health, Uganda. We also plan to disseminate these results to the general public through newspapers and online platforms.

## Authors' contribution

Authors contributed as follow to the conception or design of the work; the acquisition, analysis, or interpretation of data for the work; and drafting the work or revising it critically for important intellectual content: NS contributed 40%; GW contributed 10%, NM, BS, AN, LKS, OR, SEK, VNN, RA, RN, and JK contributed 5% each. All authors approved the version to be published and agreed to be accountable for all aspects of the work.

## Declaration of competing interest

The authors declared no conflicts of interest.
